# The Integration of a Dual-Wavelength Super Pulsed Diode Laser for Consistent Tissue Ablation in the Esthetic Zone: A Case Series

**DOI:** 10.1155/2020/8883156

**Published:** 2020-12-04

**Authors:** Nur Hafizah Kamar Affendi, Rohana Ahmad, Farhad Vahidi, Mohd Zulkifli Hassan, Siti Nadia Rahimi

**Affiliations:** ^1^Unit of Prosthodontics, Centre of Restorative Dentistry Studies, Faculty of Dentistry, Universiti Teknologi MARA, Sungai Buloh Campus, Jalan Hospital, 47000 Sungai Buloh, Selangor, Malaysia; ^2^Department of Prosthodontics, New York University, College of Dentistry, 205 E. 64th St., Ste 403, New York, NY 10065, USA; ^3^Universiti Kebangsaan Malaysia, Jalan Raja Muda Abdul Aziz, 50300 Kuala Lumpur, Federal Territory of Kuala Lumpur, Malaysia; ^4^Faculty of Dentistry, Islamic Science University of Malaysia (USIM), Malaysia

## Abstract

**Introduction:**

A diode laser is one of the universally compact accepted laser systems used fundamentally for soft tissue applications. Most diode laser devices have a single wavelength of either 810 nm for superior coagulation or 980 nm for tissue ablation. In these case series, the use of dual wavelengths (810 nm and 980 nm) in combination with super pulsing has provided a cleaner cut (no charring) with faster healing that eases the placement of the final restoration in the esthetic zone. *Case Description*. The present case series describe four cases in the esthetic zone that achieved hemostasis ablation without collateral damage to enhance gingival balance of definitive restoration. The gingivoplasty and gingivectomy modes are used to achieve efficient tissue ablation. Although there is no specific mode indicated in the FDA laser requirement for gingival depigmentation, the procedure could be safely performed with the dual-wavelength diode laser

**Result:**

All four patients revealed a good esthetic outcome and reported no pain postoperatively. Healing was uneventful, and definitive restoration was delivered within two to four weeks postoperatively.

**Conclusion:**

Within the limitation of these case series, the dual-wavelength super pulsed diode laser has the capacity to deliver peak powers resulting in efficient cutting and less tissue charring and also as an alternative tool for removal of gingival pigmentation. Prospective clinical research with larger sample size is needed for conclusive results.

## 1. Introduction

Lasers have been widely used in dentistry since the 1980s as an adjunct therapy for the removal of pathologic lesions and for esthetic procedures [1]. One of the universally compact accepted laser systems is the diode laser that is primarily used for soft tissue applications. The near-infrared (NIR) wavelength that radiates from this laser is absorbed by chromophobes (mainly hemoglobin and melanin), which produces hemostatic ablation of the targeted tissue [2, 3]. The NIR produces efficient cutting that seals well-vascularized tissue resulting in low morbidity and excellent healing as compared to traditional scalpel and electrocautery [4, 5]. Dry field surgery is permitted, and suturing is usually not necessary, resulting in an improved treatment outcome and patient comfort [6].

Nevertheless, the slow cutting effect as a result of the limitation associated with the watts and single wavelength used has been a major concern of dental practitioners. To overcome the cutting inefficiency, a “continuous wave” was used instead to provide constant energy, but this will inevitably produce collateral damage to the soft tissues [7]. This collateral damage includes tissue necrosis and delayed wound healing, which affect the tissue management in the esthetic zone. Therefore, a new generation of a diode laser with a super pulsed mode was developed to achieve efficient cutting with low heat output and decreased collateral damage [8]. The beam is modulated with high energy levels (up to 20000 Hz) with very short pulses in milliseconds for optimum clinical results [9–11]. Additionally, the incorporation of dual wavelengths of 810 and 980 nm in one unit has enhanced the coagulative and cutting efficiency which translates into cleaner cut, less charring, and better soft tissue management [11, 12]. Therefore, the purpose of these case series was to demonstrate excellent tissue management with super pulsed dual-wavelength diode lasers, which provide consistent ablation to create gingival harmony in the esthetic zone.

## 2. Methods

These case series were based on four patients who were referred to the prosthodontics department for hard and soft tissue aesthetic management of their maxillary anterior teeth. For soft tissue management, minimally invasive excision of gingival tissue was performed with a super pulsed dual-wavelength diode laser of 810 and 980 nm (Gemini Ultradent, USA), using the parameters listed in Table 1. The surgical procedure with the diode laser and its possible complications were explained to the patients, who then gave their consent. The operator, patient, and assistants wore safety glass to protect their eyes and avoided the use of mirrored surface instruments to prevent laser beam reflections. The fiber tip was initiated with a carbon paper provided in the package. The surgical area was first dried, and gingivectomy performed with a maximum peak of 2 W, in a super pulsed mode under local anaesthesia (xylocaine 2% with adrenaline, 1 : 200000). The tip was cleaned regularly with damped gauze (in normal saline) to remove any tissue debris. No periodontal dressing was used and no medication prescribed. Postoperative healing was uneventful, and no pain was reported. The final restorations were delivered within two to four weeks after surgery.

### 2.1. Case 1

**Figure 1:**
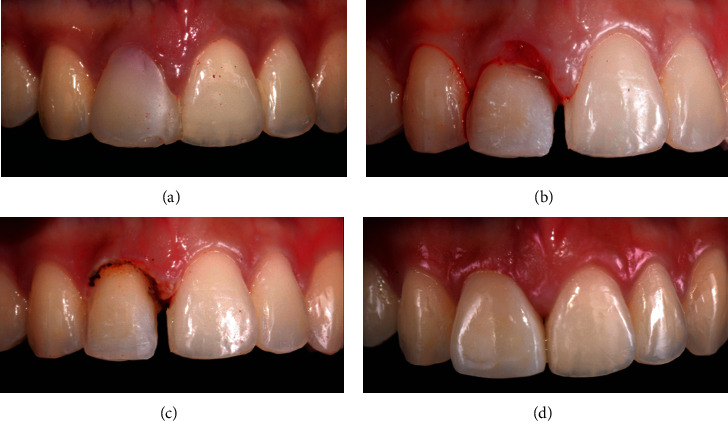
(a) Right lateral incisor in the position of the central incisor and built up with composite resin. (b) Gingival inflammation underneath the overhanging composite restoration. (c) Gingivoplasty with a diode laser. (d) Cemented veneer with excellent postoperative gingival healing.

A healthy 20-year-old female was unhappy with the shape and color of her maxillary anterior tooth. She has a congenitally missing upper right central incisor that was replaced with the lateral incisor, moved orthodontically into the place and built up with composite resin to resemble a central incisor (Figure 1(a)). Although this procedure was relatively successful in closing the anterior gap, there was noticeable discrepancy in shape, color, and gingival architecture with those of the contralateral central incisor. In addition, the overhanging margin of the composite build-up has resulted in inflammation of the gingiva and the interdental papilla (Figure 1(b)). The composite resin was removed and the tooth prepared for a porcelain veneer. To ablate the excess gingival tissue, a preset gingivoplasty mode was used with pulse duration of 140 *μ*s. The remnants of the inflamed tissue were removed and conditioned with wet sterile gauze (Figure 1(c)). The final impression for the veneer was made immediately after the procedure, and the patient was recalled after 3 days. Excellent healing has occurred, and the patient reported no pain. The lithium disilicate veneer was cemented two weeks later, and the patient expressed immense satisfaction with the outcome (Figure 1(d)).

### 2.2. Case 2

**Figure 2:**
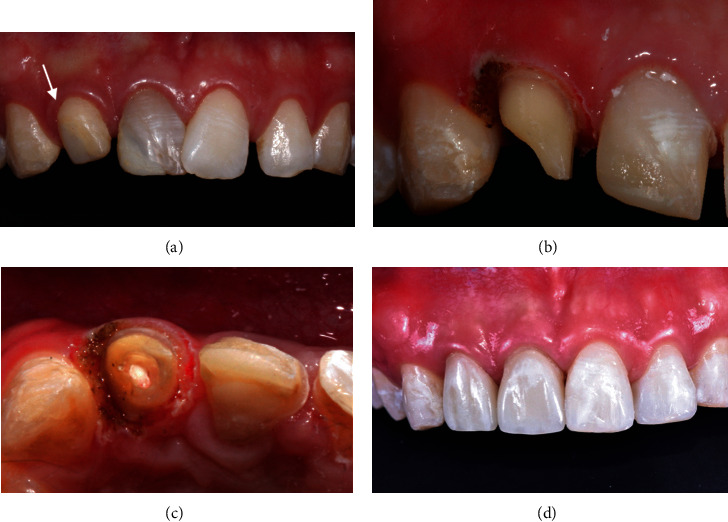
(a) Overlapping of maxillary central incisors and a peg-shaped lateral incisor built up with composite resin (arrow), (b, c) gingivoplasty on a lateral incisor, and (d) restored maxillary incisors and healed gingiva.

A young male patient complained of uneven smile and discoloration of his front teeth. Upon examination, it was noted that he has mild anterior crowding, spacing, and peg-shaped right lateral incisor restored with composite resin. The maxillary right central and lateral incisors were endodontically treated (Figure 2(a)). Various treatment options were discussed including tooth realignment with orthodontics. He declined orthodontic treatment and opted for partial and complete coverage restorations. The maxillary right central and lateral incisors and maxillary left central incisor were prepared for crowns and veneer, respectively. Gingivoplasty was then performed with a diode laser on the lateral incisor to gain tooth structure for a ferrule effect (Figures 2(b) and 2(c)). Impression was made immediately after gingivoplasty, and no periodontal dressing was necessary. Uneventful healing occurred after 5 days. The patient was ecstatic of the final outcome (Figure 2(d)).

### 2.3. Case 3

**Figure 3:**
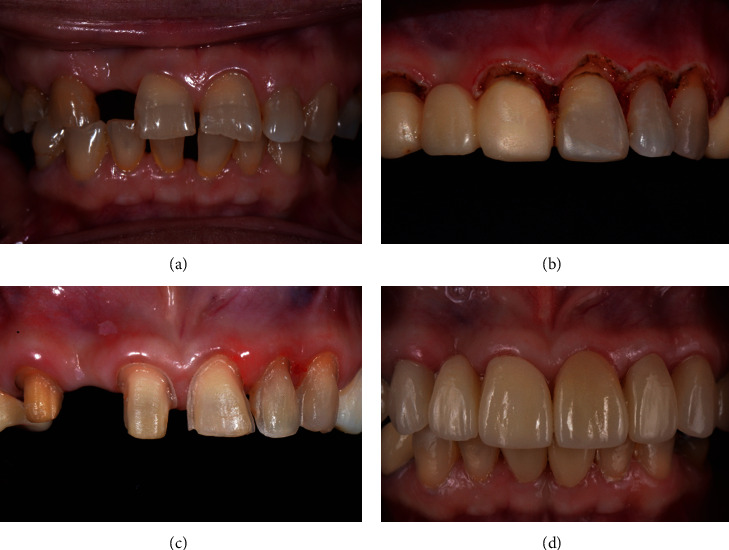
(a) Moderately worn down maxillary anterior teeth and missing a right lateral incisor. (b) Gingivectomy performed with the diode laser. (c) Final tooth preparation on healed gingiva. (d) Final prosthesis cemented showing good crown length and gingival harmony.

A female patient aged 54 years complained of unsightly appearance and requested prosthesis to replace her missing maxillary teeth. Clinical examination revealed several missing teeth and moderately worn down dentition resulting in shortened clinical crown and gingival contour disharmony (Figure 3(a)). Gingivectomy on the maxillary anterior teeth was planned to increase the crown height and improve the gingival contour before final tooth preparation and fabrication of a fixed prosthesis. A preset gingivectomy mode with a diode laser was chosen to correct the gingival zenith. Periodontal examination and bone sounding were first performed to ensure that the biologic width was preserved. The sulcular epithelium was removed under local anaesthesia by using the optic fiber tip at about 30-degree angle and passed along the marginal gingiva (Figure 3(b)). The postoperative event was uneventful, and healing of the gingival tissue was observed after two weeks (Figure 3(c)). The fixed prostheses were delivered a month after surgery, and the patient was satisfied with the final outcome (Figure 3(d)).

### 2.4. Case 4

**Figure 4:**
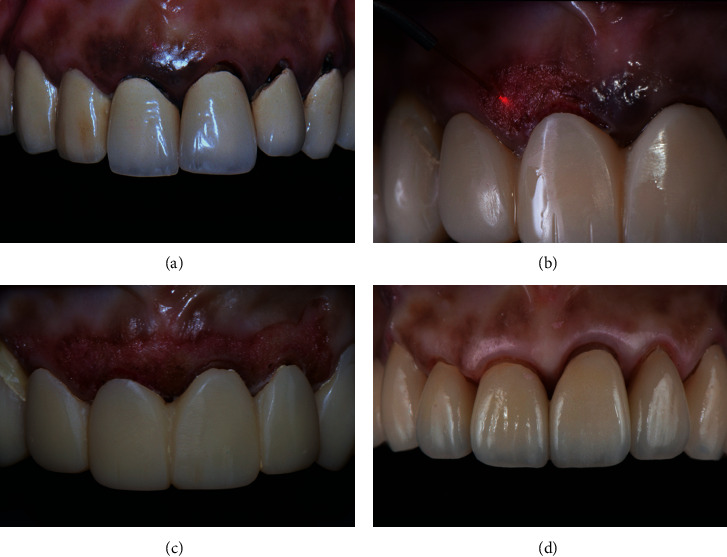
(a) Gingival pigmentation and discolored crown margins. (b) Gingivoplasty with a diode laser. (c) Immediate postoperative state of gingival peeling. (d) One-year recall showing minimal redeposition of melanin and minimal gingival recession.

A 49-year-old female patient complained of dark gingiva and corroded crown margins and requested replacement of the metal ceramic crowns (Figure 4(a)). The gingival pigmentation has a score of 3 according to the gingival pigmentation index, with diffuse brown/black pigmentation on the attached and marginal gingiva [13]. Gingivoplasty was planned to remove the pigmentation, and new all ceramic crowns were fabricated to replace the unesthetic crowns. The preset gingivoplasty mode was selected to remove the melanin pigments on the marginal and attached gingiva at the output power range of 1.0 to 2.0 watts under local anesthesia (Figure 4(b)). A depigmentation procedure was carried out on the marginal and attached gingiva at the output power range of 1.0 to 2.0 watts (Figure 4(c)). The ablation was performed with a steady brushing stroke, and the tip was kept in motion all the time. Remnants of the ablated tissue were removed using sterile gauze dampened with saline solution. Postoperative healing was uneventful after 5 days, and the crowns were cemented in a supragingival manner a month later. She was recalled after a year, and very minimal melanin redeposition was seen on the attached gingiva and the mucogingival junction (Figure 4(d)).

## 3. Discussion

Gingival health is one of the essential components that create the harmony of smile in the esthetic area. To create balance in a clinical situation which displays surplus of the gingiva, a preprosthetic gingival procedure with a diode laser has shown to be precise, sterile, and suture-less and provides superior healing [14–17]. When compared to a retraction cord for the gingival displacement technique, the diode laser is more effective and simpler to use and causes less pain [18]. As shown in cases 1 and 2, hemostatic ablations from the diode laser permit a dry field and enable gingival troughing to be done for margin accessibility and ease of impression making within the same visit. In addition, the diode laser produces wider gingival sulci, less epithelial injury, and posttreatment gingival recession as compared with the use of a conventional presaturated retraction cord prior to impression making [19, 20]. However, it has been reported that the difference in the amount of recession associated with the use of either a diode laser or a retraction cord is not significant, with an average loss of gingival height of 0.26 mm for the cord technique and 0.27 mm for the diode laser [21]. The difference was probably due to the double-cord technique applied and the single wavelength used with a continuous pulse. Hence, larger samples with more variables including tissue thickness, keratinized tissue, wavelengths, and type of pulses used are needed for a more conclusive result.

A histological study on an animal model showed that the diode laser causes more tissue damage and delayed healing as compared to erbium, chromium-doped yttrium, scandium, gallium, and garnet (Er: Cr, YSGG) and scalpel [22]. Another in vivo study has shown that histologic specimens from the diode laser cause more degenerative changes to the epithelium and stromal cells as compared to a scalpel and electrocautery in the gingivectomy procedure [17]. However, the result should be interpreted cautiously as the lateral heat may impede histologic interpretation in small lesions (<3 mm diameter) [23]. When comparing patient perception, less discomfort and pain were recorded with the diode laser [12, 24, 25], but a few studies found a higher pain value in the laser group [26, 27]. These contradictory findings could be due to the different parameters used such as wavelength, laser mode, and surgical technique. Nonetheless, as shown in case number 3, the dual-wavelength diode laser had provided efficient cutting and good bleeding control without the need for suture or dressing.

One of the remarkable advantages of the diode laser was the capability to remove a thin layer of a pigmented epithelium while preserving the connective layer and capillary vessel as shown in case 4 [28]. It was demonstrated to have a lower pain score, superior bleeding control, and less incidence of repigmentation [29, 30]. A clinical study that compared different wavelengths has found that melanin pigments effectively absorb the diode laser energy, enhance faster peeling, and cause no relapse as compared to an erbium laser. [31]. Although no specific mode for depigmentation is available in the system, it showed that this procedure could be performed safely with the dual-wavelength diode laser. Further prospective studies with a longer follow-up are necessary to confirm this “gingivoplasty” mode as one of the options to remove gingival pigmentation.

Diode laser devices have a single wavelength that ranges between 810 and 980 nm. The lower wavelength closer to 810 nm is reported to be better at coagulation, while the higher 980 nm wavelength diodes are better at tissue ablation. As documented in these case series, the combination of dual wavelengths (810 nm and 980 nm) into one unit has provided a clean cut (no charring) with uneventful healing that eases the placement of the final restoration within a month [12, 32]. While continuous and traditional pulse modes can be effective, a featured advantage of a super pulsed mode allows a more precise cut without a thermal side effect and minimal involvement of the adjacent tissue [7]. However, further studies are recommended to confirm the efficacy of the dual-wavelength approach with low laser power settings to achieve more conclusive results.

## 4. Conclusion

Within the limitation of these case series, the following conclusion could be drawn: the dual-wavelength super pulsed diode laser has the capacity to deliver peak powers resulting in fast cutting and minimal tissue charring and as an alternative tool for the removal of gingival pigmentation that was well tolerated by patients. Prospectively, it is suggested to have larger sample sizes with histological studies to explore the different impacts of wavelengths and pulse on the gingival tissue.

## Figures and Tables

**Table 1:** The parameters used for the diode laser device.

Product	Gemini
Type of laser	Diode (class IV)
Emission mode	Super pulsed
Time on/time off	Variable
Delivery system	Optical fiber
Wavelength	810 nM or 980 nM ± 10 nM
	Dual wavelength ± 10 nM (50% 810 nM and 980 nM ± 10 nM)
Peak power	2.0 watts
Average power	0.1 watt to 2.0 watts
Aiming beam power	5 mW max
Aiming beam wavelength	650 ± 10 nM
Beam divergence	617 mRad
Pulse frequency	50 Hz
Pulse width	Variable
Duty cycle	Variable
Voice confirmation	Yes
Power requirement	100-240 VAC @50 to 60 Hz–13 V

## Data Availability

The case series data used to support the findings of this study are included within the article.
